# Detecting disease associated modules and prioritizing active genes based on high throughput data

**DOI:** 10.1186/1471-2105-11-26

**Published:** 2010-01-13

**Authors:** Yu-Qing Qiu, Shihua Zhang, Xiang-Sun Zhang, Luonan Chen

**Affiliations:** 1 Academy of Mathematics and Systems Science, Chinese Academy of Sciences, Beijing, 100190, PR China; 2 Key Laboratory of Random Complex Structures and Data Science, Academy of Mathematics and Systems Science, Chinese Academy of Sciences, Beijing, 100190, PR China; 3 Key Laboratory of Systems Biology, SIBS-Novo Nordisk Translational Research Centre for Pre-diabetes, Shanghai Institutes for Biological Sciences, Chinese Academy of Sciences, 320 Yue-Yang Road, Shanghai, 200031, PR China; 4 Department of Electrical Engineering and Electronics, Osaka Sangyo University, Osaka, 574-8530, Japan

## Abstract

**Background:**

The accumulation of high-throughput data greatly promotes computational investigation of gene function in the context of complex biological systems. However, a biological function is not simply controlled by an individual gene since genes function in a cooperative manner to achieve biological processes. In the study of human diseases, rather than to discover disease related genes, identifying disease associated pathways and modules becomes an essential problem in the field of systems biology.

**Results:**

In this paper, we propose a novel method to detect disease related gene modules or dysfunctional pathways based on global characteristics of interactome coupled with gene expression data. Specifically, we exploit interacting relationships between genes to define a gene's active score function based on the kernel trick, which can represent nonlinear effects of gene cooperativity. Then, modules or pathways are inferred based on the active scores evaluated by the support vector regression in a global and integrative manner. The efficiency and robustness of the proposed method are comprehensively validated by using both simulated and real data with the comparison to existing methods.

**Conclusions:**

By applying the proposed method to two cancer related problems, i.e. breast cancer and prostate cancer, we successfully identified active modules or dysfunctional pathways related to these two types of cancers with literature confirmed evidences. We show that this network-based method is highly efficient and can be applied to a large-scale problem especially for human disease related modules or pathway extraction. Moreover, this method can also be used for prioritizing genes associated with a specific phenotype or disease.

## Background

High-throughput experimental data such as protein-protein interaction [1,2], gene expression [3], and ChIP-chip data [4], are now widely explored to study the complicated behaviors of living organisms from various aspects at molecular level. However, single type of data only provides limited information, e.g. protein-protein interaction data only tells us possible interactions among proteins rather than when and where they interact. Moreover, these data are diverse from data type to scale, e.g. protein interaction data is generally quantified as discrete values while gene expression data are usually expressed in the form of continuous values. Therefore, how to integrate these heterogeneous data to elucidate biological mechanisms is an essential and challenging problem in computational biology and systems biology. Generally, genes and their product proteins function in a concert rather than isolated manner. In particular, proteins interacting with other proteins, DNA, RNA and small molecules, form modules (e.g. complexes or pathways) to carry out cellular functions [5]. In contrast to individual components, it has been recognized that biomolecular networks or pathways are ultimately responsible to the forms and functions of living organisms, and can also reasonably explain the causes of various phenotypes. On the other hand, although protein interaction networks or pathways are available for many organisms based on accumulated protein interaction data and other experimental evidence, it is still a difficult task to identify active pathways or modules due to the changing conditions and environments in each living cell. In other words, while some genes, e.g. housekeeping genes, are constitutively expressed under various conditions to carry out basic cellular processes for growth and sustenance, most of genes or pathways are actually active only under defined conditions (e.g. at specific time and tissue). For such a problem, microarray offers a powerful tool to study gene expression patterns or active pathways under different conditions when combined with proteomic data.

The condition-specific gene analysis methods such as SAM [6] for exploring gene expression data as well as computational methods for identifying disease genes [7] have been well studied. However, these methods don't consider condition-dependent changes and cooperations among genes simultaneously. By integrating gene expression data and prior established biological knowledge, such as GO function categories [8] and KEGG pathway database [9], gene set analysis approaches, such as MAPPFinder [10], GSEA [11,12] and so on, are proposed to detect disease related differentially expressed gene sets. The principle of those methods is to rank gene sets based on enrichment of differentially expressed genes involved. Although these methods can reveal subtle but coherent gene expression changes, a major drawback is that they can not discovery new pathways correlated to phenotypes or diseases which have no records in pathway databases.

Recently, in order to identify gene modules associated to phenotypes, diseases or changing conditions, many methods [13-21] have been developed by integrating interactome with gene expression data. A disease associated active module can be considered as a connected subnetwork or dysfunctional pathway in a biomolecular interaction network which has close relationship with a specific disease. Previous works to detect an active module generally include two steps. In the first step, a scoring scheme to evaluate a module's active level is adopted based on each gene's or interaction's active level from gene expression data. The scoring function is usually designed to be an additive function of each gene's active level. Ideker *et al*. [13] first formulated the problem of the active pathway detection, where the scoring scheme is given by a summational function of all genes' differentially expressed *p*-value within the subnetwork. Dittrich *et al*. [19] used an additive function of *p*-values based on a mixture model. Breitling *et al*. [14] proposed a method named GiGA to score subnetworks in terms of genes' order of their differential expression significance. In these methods, genes in the same pathway are assumed to be independent, and the correlation or cooperativity between genes are not considered. Thus, Guo *et al*. [17] and Nacu *et al*. [16] modeled the relationship between genes in a local manner so that only neighborhood relations of genes are considered. In the second step, a search procedure is implemented to find an active module from a molecular interaction network such that the nodes of the active module are connected in the subnetwork with a highest score. Unfortunately, this procedure is an NP-hard problem as was proved by [13]. Several heuristic or approximate methods were proposed to deal with this problem, e.g. simulated annealing based [13,17], locally greedy search based [14-16,18] and mathematical programming based methods [19,21]. However, these methods typically suffer from inefficiency and inaccuracy problems due to the NP-hard nature, thereby are not tractable for practical applications.

In this paper, with the consideration of the global relationship among genes, we propose a novel network-based method to identify disease associated modules by integrating both protein-protein interaction and gene expression data in an efficient and accurate manner. The proposed method based on a regression model with a diffusion kernel is denoted as RegMOD, which not only can theoretically model the nonlinear effect of gene cooperativity but also is computationally efficient. We have tested RegMOD on both simulated and real world datasets. The results of numerical experiments demonstrate its efficiency and robustness. Furthermore, by applying to the breast cancer and prostate cancer datasets, we successfully and efficiently identified disease related active modules which have been confirmed to correspond to the known molecular mechanism of these two types of cancers. Clearly, the identified active modules display a possible scenario of dysfunctional gene cooperativity for complex diseases.

## Methods

### Principle of RegMOD method

Genes or proteins are linked to form a network by interactions, which facilitates biological functions. From the perspective of information flow, the information of biological systems contained in genes is transferred to proteins or other molecules for executing biological functions. In this paper, we do not distinguish gene with its product protein as used in the seminal paper of Ideker *et al*. [13]. The nodes in the interaction network represent genes and links between genes represent interactions including protein-protein interaction, protein-DNA interaction or other functional linkages.

**Figure 1:**
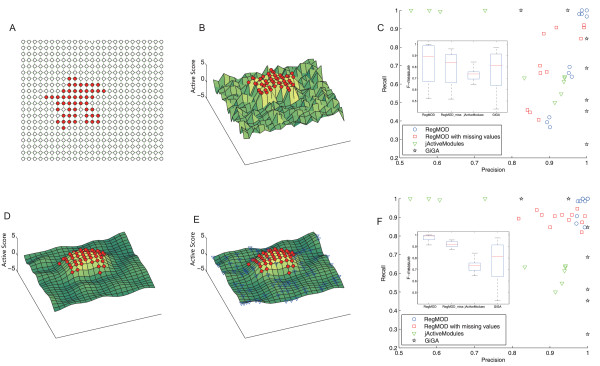
**Illustration of the effect of RegMOD**. (A) shows the grid network where the red nodes represent the active module. (B) and (D) illustrate the active score surfaces before and after the processing of RegMOD respectively. (E) shows the active score surface obtained by RegMOD when the nodes represented by blue triangle are deleted. In the randomly generated network example, the recall-precision plot and box-plot of F-measure for RegMOD are shown in (C). In the edge-weighted case, the performance is significantly improved. The recall-precision plot and box-plot of F-measure for RegMOD are shown in (F).

Based on the assumption that interacting genes have similar active scores which measure the extent to which genes respond to a specific disease. The active score of a gene is defined by a nonlinear active scoring function which considers the cooperations among genes. To estimate these underlying active scores defined by the active scoring function, a kind of observed active scores defined as differentially expressed levels of genes is calculated from the case-control microarray data. The goal is to estimate the underlying active scores for each genes which approach to the observed active scores and capture the cooperative pattern simultaneously. This is formulated as a typical regression problem by fitting the active scoring function to the observed active scores. In this paper, we use the support vector regression method with a diffusion kernel to evaluate each gene's underlying active score. This procedure can be regarded as a smoothing process that gives each gene a new active score. It can eliminate acute changes among neighboring genes in the network and infer underlying active scores of genes whose expression level could not be measured (Figure 1). It can significantly improve the accuracy and robustness of the predicted activity of genes and reduce the effect of noise and incompleteness of the high-throughput data. Finally, the induced subnetworks of significantly scored genes form the active modules which are expected to be related with a specific phenotype or disease. Particularly, prioritizing genes according to their active scores can provide the order of gene's association level with a specific phenotype or disease.

### Regression Model with Diffusion Kernel

Genes interacting with each other can be expressed as a network. The gene expression data provides valuable information on gene's activity, which can be represented by weights of genes and interactions in the network. Formally, an interaction network with weighted nodes and weighted edges can be expressed as *G *= (*V*, *E*, *S*, *C*), where node set *V *represents genes, edge set *E *represents interactions, *S *represents the scores or weights of nodes, i.e. the active level of each node, and *C *represents the weight of edges. Since coexpressed genes are more likely to function together, the edge weight is defined as absolute Pearson correlation coefficient of expression profile of two node genes as following

where *y *and *z *are two expression profiles,  and  are mean expression values. For a node subset *V' *⊆ *V*, *G' *= (*V'*, *E'*, *S'*, *C'*), where ,  and , is the induced subnetwork of *V' *from *G*.

To model the relationship between active scores and genes, we construct a function in which underlying active score *f*_*i *_of gene *i *is taken as a response variable and genes *x*_1_, *x*_2_,..., *x*_*n *_are viewed as explain variables, i.e.,

where *x*_*i*_, *i *= 1,..., *n *is the attribute vector of each gene which contains the biological information. Clearly, each gene's active score is affected by other genes. The relationships between genes are involved in this nonlinear function rather than an additive function which assumes non-cooperativity among genes. In other words, in contrast to the existing methods, the proposed model can represent cooperative effect among genes.

By a nonlinear mapping *ϕ *as in [22,23], we transform the relationship of genes and active scores from the input space of *x *into a feature space (such as reproducing kernel Hilbert space). Then, the nonlinear function *f *in the input space can be expressed as a linear function in the feature space

Based on the representation theorem [22,23], *u *can be expressed as  in the reproducing kernel Hilbert space which has good theoretical properties. Using the kernel trick as mentioned in [22,23], the inner products in the feature space can be calculated by a kernel function in the input space, i.e. *k*_*ij *_= ⟨*ϕ*(*x*_*i*_), *ϕ*(*x*_*j*_)⟩, *i*, *j *= 1, 2,..., *n*. Then

where *k*_*ij *_is the kernel function which is the similarity of gene *i *and gene *j*. Thus, it is unnecessary to know neither the mapping *ϕ *nor the feature space exactly due to the kernel trick. In other words, the transformed nonlinear function can be numerically evaluated simply by a linear mode, thereby greatly simplifying computation. Evaluating the kernel function on all pairs of genes yields a kernel matrix *K *= (*k*_*ij*_), *i*, *j *= 1, 2,..., *n*, which is symmetric and positive semi-definite. There are many kernels such as Gaussian kernel, polynomial kernel and spline kernel to describe the similarity of continuous variables. To model similarity of nodes in a network which has a discrete structure, the following diffusion graph kernel from [24] which simulates the heat transduction in the network is adopted [23],

where *τ *controls the magnitude of the diffusion and *L *is the graph Laplacian matrix

where  is the degree of node *i*. For edge-unweighted case, *C*_*ij *_= 1 if node *i *and *j *are adjacent and *C*_*ij *_= 0 otherwise. Note that even for an edge-weighted network, its diffusion kernel can be calculated in the same way. *k*_*ij *_describing the similarity of two nodes increases with the shortest-path distance and the number of all possible paths between them [24]. Thus, with diffusion kernel, *f *is calculated based on nodes' topological similarity in the network.

In the following part, we introduce how to define the observed active score for each gene. For a microarray dataset including two classes of samples, e.g. case and control, the observed active score of each gene is evaluated by the Signal to Noise Ratio (SNR). For the *i*th gene, its expression profile can be divided into two classes and its SNR active score can be calculated as

where *μ*_*i*1 _and *μ*_*i*2 _are the means of the expression levels of gene *i *in sample set 1 and sample set 2 respectively, and *σ*_*i*1 _and *σ*_*i*2 _are the standard deviations of gene *i *in sample set 1 and sample set 2 respectively. There are only a few number of samples contained in the microarray data which can hardly meet the statistical significance, therefore this simple metric SNR is more compatible and has been successfully used to detect disease related genes and gene sets [11,25]. Other statistics such as t-statistics and statistics used in SAM [6] have also been tested. SNR performances better than other statistics, thus we adopt this metric for further analysis (See Figure S2 in the Additional file 1).

The SNR score of each gene *w*_*i *_is regarded as the observed value of underlying active score *f*_*i*_. Due to the noise and incompleteness of the microarray data, the observed active score may not well reflect the fact that interacting genes have similar active scores. Thus, using *f *to fit the observed score can predict the underlying active score which captures the cooperative patterns among genes and is most close to the observation. In practice, the fitting process is to estimate the parameters *β *in function *f *towards *w*_*i*_. This forms a nonlinear regression problem, which can be solved by the following support vector regression (SVR) [22] model,

where  is the regularization term and C* is a regularization constant, they control the complexity of *f *and prevent from overfitting. This problem is solved in its dual space by converting the primal problem to its dual problem which is a convex quadratic programming:

where *α*_*i *_and  are Lagrange multipliers, *β*_*i *_= (*α*_*i *_- ), *ε *is a small constant. Thus, the globally optimal solution can be obtained even for a large scale problem. In this study, the widely used software LIBSVM from [26] was employed to solve the SVR problem.

After *f *is fitted, each gene gets a new active score *f*_*i *_which is regarded as the underlying active score. Since the observed active score is noisy and has many outliers, the proposed method RegMOD can smooth out the outliers and estimate the underlying active score by integrating the network topological similarity between genes. A gene with a low *w *score but functioning as a bridge to connect high weighted genes will get a newly high *f *score. Conversely, a gene with a high *w *score but interacting with low score genes will get a newly low *f *score. The Matlab code of RegMOD is available in the Additional file 2.

Based on the estimated underlying active score, we extract the active modules from the network. The high scoring region in the network constitutes active modules which associate to the specific phenotype or disease. To extract these modules, the high scoring active gene set including up-regulated gene set *VU*_*f *_and down-regulated gene set *VD*_*f*_, are selected as follows,

where  is the median value of *f *and *σ *is the median absolute deviation of *f*, *θ *represents the fold change apart from the median. Genes with *f *value higher than the fold change are considered significantly active. In this paper, we found that *θ *= 6 is reasonable for obtaining biologically significant results in both practical applications (See Figure S3 in the Additional file 1). Then, we can obtain the induced subnetwork *GU*_*f *_(*GD*_*f*_) of *VU*_*f*_(*VD*_*f*_) from the interaction network. The connected components of *GU*_*f *_(*GD*_*f*_) correspond to the up-regulated (down-regulated) modules.

### Performance measurements

To evaluate the performance of the accuracy of the identified active modules in the simulated dataset, the recall and precision (denoted as *r *and *p *respectively) are applied and defined as below:

The F-measure which is the harmonic mean of precision and recall is used to measure total accuracy:

Large F-measure value indicates good performance of the results. For each identified module by one method, we calculate the *r*, *p *and F-measure. The recall-precision plot and box-plot of F-measure are implemented to compare the performance of different methods.

To measure the topological similarity of two genes within a module, the following index is adopted:

where *k*_*ij *_is the element of the diffusion kernel matrix *K*. The logarithmic transformation could facilitate to visualize subtle changes. Other logarithm base, such as 2 or *e*, could also be used, which do not affect the results. The high value of similarity indicates tight or close relationship between two genes.

**Figure 2:**
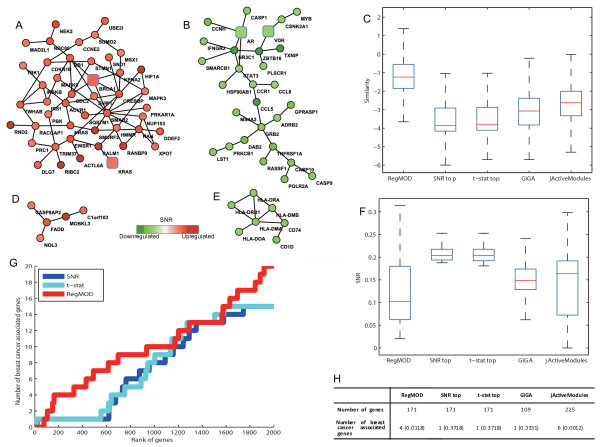
**Breast cancer metastasis associated modules identified by RegMOD**. The square nodes refer to known breast cancer related genes. (A) and (D) are up-regulated modules BCUM1 and BCUM2 which are related to cell cycle and apoptosis respectively in red color. (B) and (E) are down-regulated modules BCDM1 and BCDM2 which are related to signaling transduction and antigen presentation respectively. (C) and (F) chart the box-plot of the similarity among genes and SNR values of genes involved in active modules found by different methods. The distributions of breast cancer genes on different gene ranking lists are shown in (G). (H) charts the comparison of gene sets' coverage of known breast cancer associated genes using different methods with the significant p-values calculated by hypergeometric distribution.

**Figure 3:**
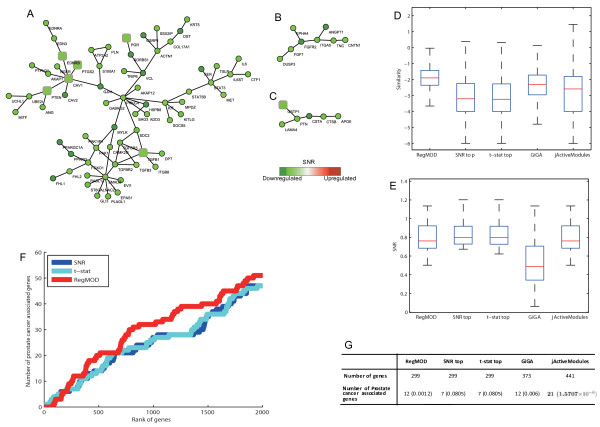
**Prostate cancer associated modules identified by RegMOD**. The square nodes refer to known prostate caner related genes. (A), (B) and (C) are three down-regulated modules PCDM1, PCDM2 and PCDM3 which are related to cell adhesion, receptor binding and cell growth respectively. (D) and (E) chart box-plot of the similarity among genes and the SNR of genes involved in active modules found by different methods. (F) chats the distribution of prostate cancer genes on different gene ranking lists. (G) charts comparison of modules' coverage of known prostate cancer related genes using different methods.

Prioritizing genes according to the absolute active scores can generate a gene ranking list. The distribution of disease related genes in this ranking list is used for evaluating the active score's biological meaning. The plot of coverage of disease genes versus the rank of genes is used (See Figure 2G and 3F, Figure S2, S3, S4 and S5 in the Additional file 1), i.e. each point in the figure represents the number of disease related genes in the set of genes with higher rank than a value.

## Results and Discussion

### Materials

We first tested our method on two simulated networks. The first one was a grid network with 500 nodes (Figure 1A) which was designed for better visualization. A connected subnetwork with 50 nodes was randomly selected from the whole network to represent an active module. Firstly, a seed node was selected as an initial active subnetwork from all nodes of the network. Then, one neighbor node of the active subnetwork was randomly selected to be added to the current active subnetwork. This add-in process was repeated until the subnetwork size reached to a previously defined value (e.g. 50 in this example). As the same setting of [15], each node in the active modules was given an active significance *p*-value uniformly distributed between 0 to 10^-3^, and other nodes were given *p*-values uniformly distributed between 0 to 1 representing the background activity. Since the *p*-value assigned to each node cannot be transformed to a SNR score, it was transformed to a *Z*-score to represent active score by an inverse cumulated standard normal distribution function as used by [13]. The edge weights were all set to 1. To test RegMOD on general network, another network of 500 nodes with randomly generated topology structure was used. An active connected subnetwork of 50 nodes as well as the active scores was also generated as described in the first example. Two types of edge weights were tested in this example. For the edge-weighted case, edges within the active module were weighted by scores uniformly distributed in (0.5, 1) and others were weighted by scores uniformly distributed in (0, 1), to simulate the correlation of gene expression profiles. For the edge-unweighted case, the weights of all edges are set to 1.

The proposed method was applied to study two real biological problems. The molecular interaction network was from the HPRD [27] protein-protein interaction database (release 7) which contains 9460 genes with 37083 interactions. Two gene expression datasets were used to test our method. One of them was generated by breast cancer tumor gene expression experiments [28], which contains gene expression profiles for 286 patients with lymph-node-negative primary breast cancer. No patient received any adjuvant therapy. During the follow-up period, 180 of these patients were relapse-free at 5 years, and 106 of them developed distant metastasis. The other gene expression dataset [29] of prostate cancer was from profiling 74 tumor and 41 normal prostate specimens samples using cDNA microarrays. All expression data were downloaded from the SMD [30] database. We preprocessed the data in a popular procedure used by many studies [17,31]. Probes with missing data in more than 10% of arrays were screened out. Then, we applied base-2 logarithmic transformation and carried out data normalization so that the expression values had the mean 0 and standard deviation 1 in every array. Genes' expression profiles were mapped from probe annotation. For the gene measured by many probes, the mean expression value was used. Finally, profiles of genes which were contained in the network were used for further analysis. There were 7948 genes in the network which had expression profiles in the breast cancer dataset, and 6265 genes in the network were profiled in the prostate cancer dataset.

A list of 60 breast cancer susceptibility genes (52 of them are present in the network) collected by [31], which included 13 genes recorded in OMIM [32], was used to evaluate a module's coverage of the disease genes. For the prostate cancer dataset, a list of prostate cancer related genes included in the Prostate Gene Database (PGDB) [33] and recorded in OMIM [32] was used. There were totally 183 prostate cancer related genes in which 140 genes were present in the network.

In further analysis, the Molecular Signatures Database (MSigDB) [12], which was a collection of gene sets including known, experimental and computational sets of genes with common properties such as involvement in the same canonical pathway or GO category, was used to annotate identified modules. The best overlapping gene set in the database with an identified module was provided as annotation based on *p*-value computed by hypergeometric distribution. Here, two types of gene sets with known biological function, canonical pathway and GO category, were used. The significant overlapped gene sets were ranked by the *p*-values and the first 20 significant sets such that *p*-value < 0.05 were selected as annotations.

Two methods jActiveModues [13] and GiGA [14] were also applied to the same datasets for comparison. The jActiveModules which is a plug-in of Cytoscape [34] implements the method proposed by [13]. The jActiveModules assigns a Z-score which measures the differentially expressed level of a gene to each gene and searches active subnetworks using two alternative strategies: the simulated annealing and greedy search. In this paper, the *p*-values used in jActiveModules were calculated via *t*-test. Another method GiGA assigns the rank of genes based on the differentially expressed level to each gene. It finds active subnetworks by iteratively extending subnetworks which are initially from the local minimal nodes. The gene order lists used in GiGA were ranked in terms of SNR by ascending or descending order to find up- or down-regulated modules.

### Results on Simulated Dataset

We performed 20 different runs to test the performance of the proposed RegMOD method and all the different runs showed very consistent results. Here, for clearly showing the effect of parameters and comparing with other methods, we randomly selected one network for each testing example. To illustrate the visual effect of RegMOD method, we first assessed our method on a grid network with a predefined active module (as shown in Figure 1A). If one regards the score of each node as the height and connects them with a continuous surface, it constitutes a ridgy surface (called active score surface) above the network as shown in Figure 1B. The active module is composed of some peaks of the surface. After applying the proposed RegMOD, the active score surface in Figure 1D became smooth and the active module was more clear than that in Figure 1B where it was present as a unique peak of the surface. We randomly deleted 100 nodes from the network and then estimated the active score surface. As illustrated in Figure 1E, the surface was almost the same as the one without missing nodes.

More quantitatively, we tested our method on another randomly generated network (as described in Materials) to detect the predefined active module. Different parameters (*τ *= {1, 2, 3,4, 5} and *θ *= {2, 4, 6}, totally 15 different combinations) were set, and corresponding recall, precision and F-measure were calculated. To compare with existing methods, we ran the software jActiveModules 10 times with the simulated annealing strategy based on different well tuned parameters, and evaluated results in the same way. The GiGA was also applied to this data set with different parameter *m *(*m *= 40 to 60, which is around the true active subnetwork size 50). Deleting part of nodes from the network randomly, the proposed RegMOD was also tested. The recall-precision plot of results (see Figure 1C) showed that the active module was successfully identified by RegMOD in some cases even with missing values. We found that jActiveModules could hardly get the results which perform well in both recall and precision. The GiGA had better performance than jActiveModules but was worse than RegMOD. The box-plot of F-measure of the results (see Figure 1C) indicated that average performance of RegMOD was superior to other methods.

Considering the case of weighted edges (as described in Materials), the performance of the proposed method was significantly improved, comparing with unweighted case (see Figure 1F). The most of identified modules achieved high recall and precision in all cases even with missing values. The average F-measure of RegMOD was high and the variance among different results was small. Since jActiveModules and GiGA did not use the information of edges, the performance was still the same as previous results. Comparing with these two cases, clearly, RegMOD achieved high accuracy in the edge-weighted network, and the performance was also robust to parameters, noises and missing values. In the following real biological applications, we tested the RegMOD to detect modules and genes related to cancers in weighted networks which incorporate the correlation of gene expression profiles into the interaction network.

### Results on Breast Cancer Dataset

Since two highly co-expressed genes are more likely to interact together to facilitate biological functions, we tested the RegMOD on the edge-weighted interaction network by integrating the Pearson correlation coefficient value of two genes' expression profile, and the diffusion kernel parameter *τ *= 3 and regularization factor C* = 1 (sensitivity analysis showed in Figure S3 and S5 in Additional file 1). Ranking the gene's absolute active score *f*, a gene list was given. In the first 2000 genes, the breast cancer related genes were enriched in this list comparing with other lists ranked based on absolute value of SNR and *t*-statistic (denoted as *t*-stat) (see Figure 2G). By setting *θ *= 6, we got a compact gene list with 171 genes. BRCA1, KRAS, VDR and AR which are breast cancer associated genes were presented in this list. Its induced subnetwork contained 2 up-regulated modules (denoted as BCUM1 and BCUM2) and 2 down-regulated modules (denoted as BCDM1 and BCDM2) according to the activity changing directions of genes (see Figures 2A, B, D and 2E). Figures 2C and 2F show that the SNR values of genes in modules identified by RegMOD were slightly lower than modules by other methods while the similarity among genes was higher.

We further investigated modules based on the annotation by MSigDB. One up-regulated module BCUM1 (Figure 2A) included 52 genes among which there are two breast cancer related genes BRCA1 and KRAS. This module is a cell cycle related module which is enriched with cell cycle related genes (cell cycle GO terms overlapping *p*-value = 2.53 × 10^-11 ^and cell cycle pathway overlapping *p*-value = 8.86 × 10^-7^). Many studies indicate that cell cycle and proliferation genes are associated with higher grade, poor prognosis tumors (see [35] and the references therein). The module is also enriched with genes associated with membrane progesterone receptor pathway (*p*-value = 5.35 × 10^-5^), and ERBB signal pathway (*p*-value = 1.13 × 10^-3^). Progesterone signal plays an important role in breast cancer development and progression [36], and progesterone receptor signaling has a role in breast cancer cell movement and invasion through the actin cytoskeleton [37]. ERBB2 is a well-established prognostic marker, and the signaling processes driven by ERBB receptors are related to breast cancer [38]. Another up-regulated module BCUM2 (Figure 2B) is an apoptosis related module which is enriched with apoptosis GO function (*p*-value = 5.35 × 10^-4^). We found that it significantly overlaps with the Fas signaling pathway which induces apoptosis and NF-kB activation [39]. Dysregulated apoptosis contributes to malignant progression in breast cancer [40].

In the two down-regulated modules, BCDM1 (Figure 2D) is a signal transduction related module which contains 33 genes including two breast cancer related genes AR and VDR. It enriches with signal transduction and related GO function terms, and significantly overlaps with Interleukin-6 signal pathway (*p*-value = 6.38 × 10^-6^), apoptosis pathway (*p*-value = 1.05 × 10^-5^), epidermal growth factor signal pathway (*p*-value = 1.81 × 10^-5^) etc. which are related to cell grow, inflammation, proliferation, and apoptosis signaling transduction. Previous study of [41,42] pointed out that dysregulation of growth signaling transduction molecules resulted in uncontrolled proliferation and survival, ending in tumor initiation and progression. The other down-regulated module BCDM2 (Figure 2E) is an antigen presentation related module containing 8 genes where 7 of them were involved in antigen processing and presentation pathway (*p*-value = 1.26 × 10^-12^). This confirms the fact that in patients with advanced-stage breast cancer, antigen presentation was decreased [43,44].

By ranking absolute SNR and t-statistic, both two of lists for the top 171 genes contain one breast cancer associated gene. GiGA was also applied to these data, and the identified 4 significant active subnetworks covered only one breast cancer associated gene. The jActiveModules was used to find active subnetworks for comparison, and obtained 3 active subnetworks. The results include 6 breast cancer related genes. The *p*-value of coverage significance indicates that our method is able to efficiently extract compact gene list with a significant high coverage for breast cancer related genes (See Figure 2H). Although subnetworks found by jActivemodules also covered many breast caner associated genes, the subnetwork is too large to be interpreted, and do not distinguish the up- and down-regulated modules. The absolute SNR value of each gene in the active modules identified by different methods is shown in Figure 2F. The absolute SNR of genes in the modules identified by RegMOD is a little lower than in other modules. However, the similarity among genes is higher than others (see Figure 2C) which means that the identified modules containing genes with high correlation are more likely to achieve particular functions.

We further analyzed modules identified by Reg-MOD and other methods in terms of the overlap of known pathways and GO categories enrichment(shown in Tables S1 and S2 in Additional file 1). The down-regulated module BCDM2 (Figure 2E) is highly overlapped (6 overlapped genes) with one down-regulated module (18 genes) identified by GiGA and one active subnetwork (6 genes) identified by jActiveModules. The six genes in the three modules are all involved in antigen processing and presentation pathway. The pathways overlapped and enriched GO categories are almost the same. This indicates that antigen processing and presentation are inhibited in metastasis tumor. The down regulated module BCDM1 and one module identified by GiGA overlap with 6 common pathways and enrich with 8 common GO terms which are related to signal transduction. However, there are some differences between them, for example, Interleukin-6 signal pathway and epidermal growth factor signal pathway which are related to breast cancer are present in the pathway overlapping list of BCDM1 but missed in the other. The pathway overlapping and GO category enrichment list of up-regulated module BCUM1 is highly similar to one module identified by GiGA and one by jActivemodules. The three modules all contain BRCA1 and were highly related to cell cycle. However, some differences still exist, such as, ERBB signaling pathway which is associated to breast cancer is present in the BCUM1 pathway overlapping list while absent in other lists.

### Results on Prostate Cancer Dataset

In another case study of prostate cancer, we applied RegMOD with *τ *= 3 and regularization factor C* = 1 (sensitivity analysis showed in Figure S4 and S5 in Additional file 1) to the network weighed by Pearson correlation coefficient of two genes' expression profile. The ranking list of genes in terms of the estimated underlying active score *f *enriched with the prostate cancer related genes comparing with other ranking methods (see Figure 3F). By setting *θ *= 6, a list of 299 genes including 12 prostate cancer related genes were selected for further analysis. The induced subnetworks contain 3 down-regulated modules (denoted as PCDM1, PCDM2 and PCDM3, which contain 69, 8 and 6 genes respectively) as illustrated in Figures 3A-C. These modules both have high intra-module similarity and differentially expressed levels as showed in Figures 3D and 3E. PCDM1 (see Figure 3A) is a cell adhesion related module which contains 6 prostate cancer related genes. It is significantly overlapped with the cell adhesion related pathway such as, adherens junction pathway (*p*-value = 3.35 × 10^-6^) and focal adhesion pathway (*p*-value = 1.43 × 10^-4^). The GO annotation indicates that it is enriched with cell achesion related GO terms, such as cell substrate adherens junction (*p*-value = 6.84 × 10^-6^) and adherens junction (*p*-value = 3.15 × 10^-5^). Cell adhesion is a hallmark of prostate cancer cells. E-cadherin, N-cadherin, *β*-catenin, integrins, focal adhesion kinase, connexins and matrix metalloproteinases all appear to be promising biological markers associated with the early stage metastatic process in prostate cancer [45]. Another module PCDM2 (Figure 3B) is related to receptor binding. It enriches with genes of receptor binding GO annotation and those genes involved 4 pathways such as regulation of actin cytoskeleton, MAPK signaling pathway. Several groups have found evidence that the MAPK signaling pathway is highly associated with prostate cancer [46]. The third module PCDM3 (Figure 3C) containing one prostate cancer related gene GSTP1 is related to cell growth. It is enriched with GO terms related to cell apoptosis, death and development. Dysregulation of these processes contributes to tumor progress and metastasis [46].

Other methods were also applied to this dataset. The resulted modules' coverage of prostate cancer related genes is showed in Figure 3G. Ranking genes based on the SNR and *t*-statistics (denoted as *t*-stat) and selecting the top ranked 299 genes, we obtained a gene list containing 7 prostate related genes. The GiGA found 373 genes involving 4 active modules and covering 12 prostate related genes. The jActive-Modules found an active module of 441 genes which contains 21 prostate related genes. Comparing with these methods, the gene list identified by RegMOD is more compact than GiGA and jActiveModules, and covers more prostate cancer related genes than ranking SNR or *t*-statistics and GiGA, thereby verifying the effectiveness and efficiency of our method.

Further investigation of the annotation of modules identified by RegMOD and other methods is shown in Table S3 and S4 (see Additional file 1). PCDM1 has a similar GO and pathway annotation with two down-regulated modules found by GiGA and one active module by jActiveModules. However, some annotations are unique in PCMD1, such as renal cell carcinama pathway, TGFB pathway and nuclear import GO terms. The TGF beta signaling (TGFB) pathway is related to regulation of growth and proliferation of cells which are also related to tumor progress [46]. The pathway annotations of PCDM2 are involved in one module found by GiGA and the active module found by jActiveModules while the GO annotation is not. The GO annotations of PCDM3 are diverse from other modules. As analyzed above, these GO annotations related to cell growth are also related to tumor progress.

## Discussion

Microarray data and interactomic data play key roles in the pathway extraction and analysis. Microarray technology facilitates the identification of genes involved in the human diseases by providing disease specific gene activity information, whereas the inter-actomic data shed light on the probability of genes and proteins interacting with each other. Assembling these two types of data can uncover disease associated genes, pathways and molecular mechanisms. In this paper, we proposed an efficient and robust method to identify disease associated modules, which can be pathways, complexes or crosstalk subnetworks between pathways related to the disease process. Specifically, from a systematic perspective, the relationship between genes and a specific disease is modeled by a nonlinear active scoring function in this paper. Then, genes' active scores which measure the level of their responses to the disease are defined by a diffusion kernel, which represent cooperative effect of genes. The active scoring function is estimated by the support vector regression method which is efficient even for a large-scale problem. Finally, the disease related modules are derived from the high ranking genes. From computational viewpoint, our method can solve large scale problems in the global optimization sense, in contrast to the conventional heuristic methods. From the numerical example, we show that our method achieves high performance and is superior to other methods by identifying modules with compactness and high coverage of disease related genes. Applying our method to breast cancer and prostate cancer data, we identified compact breast and prostate cancer related modules confirmed by literatures. Note that this method also provides a gene ranking list which is useful for choosing the closely related genes for further experimental verification. In addition, we can get the active modules with different sizes which are nested each other by altering the threshold from high to low. This hierarchical structure which is similar to the contour map is easy to be used for exploring the distribution of the active region in the whole network.

The condition-specific pathway identification and gene function prediction are two typical problems in bioinformatics. Here we introduce RegMOD to address the first problem based on the assumption that interacting proteins have similar activity. A related computational framework employing Markov random field model [47] and diffusion kernel with the support vector machine [48] for protein function prediction have been proposed recently. The underlying rationale is that proteins with similar function annotation are more likely to interact with each other. Furthermore, the methodologies are different since the protein function prediction problem uses classification method, while RegMOD employs a regression model. In the numerical and real world examples, we prove that RegMOD is an effective method for gene active pattern discovery, the disease associated pathway identification and genes inference.

The parameters used in the RegMOD are selected empirically in terms of the topology of the network and correlation between genes. In the future, we will further refine the model selection method to improve the computational efficiency. In addition, many sources of data, such as mass spectrometry, SNP and clinical outcome data, can be assembled into this model to achieve high accuracy. For instance, by describing these data as kernel matrices, we can combine them by an additive function of kernel matrix, thereby integrating different data sources efficiently. Another factor affecting the performance of RegMOD is incompleteness of the interaction data. While, the identified module is largely restricted by the topology of the network. One proper way to address this problem is to integrated other molecular interaction data, such as protein-DNA interactions or protein-compound interactions, with protein-protein interaction data to construct a network as [49] including signal transduction, gene transcription and metabolic network. On the other hand, along with deeper research of biological system, more and more interactions will be verified by experiments, which will promote the performance of RegMOD.

## Conclusions

In conclusion, a novel method is proposed for detecting disease associated modules by integrating protein-protein interaction and gene expression data. Different from existing methods, the cooperations of genes are considered. The testing results on artificial dataset and real world results indicate that it is robust to noise and missing values and identifies disease associated modules with literature confirmed evidences. Due to computationally efficiency, it can be applied for large scale problems. In addition, the ReMOD method uncovers each gene's active scores which facilitate disease associated gene ranking.

## Authors' contributions

YQQ proposed the original idea, YQQ and SZ designed the details and implemented the experiments, LC and XSZ improved the methods and give valuable suggestions. All authors wrote and approved the manuscript.

## Supplementary Material

Additional file 1 Supplementary Materials Click here for file

Additional file 2 RegMOD code Click here for file
