# Supplementary material for: Detecting disease associated modules and prioritizing active genes based on high throughput data
Source: BMC Bioinformatics. 2010 Jan 13;11:26. doi: 10.1186/1471-2105-11-26 (PMC2825224; doi:10.1186/1471-2105-11-26)
Supplement: Additional file 1 — Supplementary Materials [file 1471-2105-11-26-S1.PDF]

Supplementary Materials

1 Active score comparison

SAM and t-statistic are also used as observed active score metric for predicting the real active score of each gene. We first compare the correlation of different scores. All genes' active scores compose to a vector. The observed active score vector calculated by t-statistic, SAM and SNR are denoted as t-stat, SAM and SNR respectively, and the corresponding predicted underlying active score vector are denoted as t-stat_pre, SAM_pre and SNR_pre respectively. Then, the Pearson correlation coefficient (PCC) are used to evaluate the relationship between these score vectors (See Figure S1). High PCC value indicates high similarity between score vectors. In the both case studies, the observed score vectors (t-stat, SAM and SNR) are with high similarity and predicted score vectors (t-stat_pre, SAM_pre and SNR_pre) are also similar to each other, while the similarity between observed scores and predicted scores are low. The predicted active score using SNR metric is a little different from other predicted active score. However, in the ranking list of genes based on active scores, the disease related gene enrichment is higher than the ranking list based on other active score (See Figure S2). It is noticed that the predicted underlying active score ranking list (t-stat_pre, SAM_pre and SNR_pre) enrich of more disease gene than observed active score ranking list (t-stat, SAM and SNR).

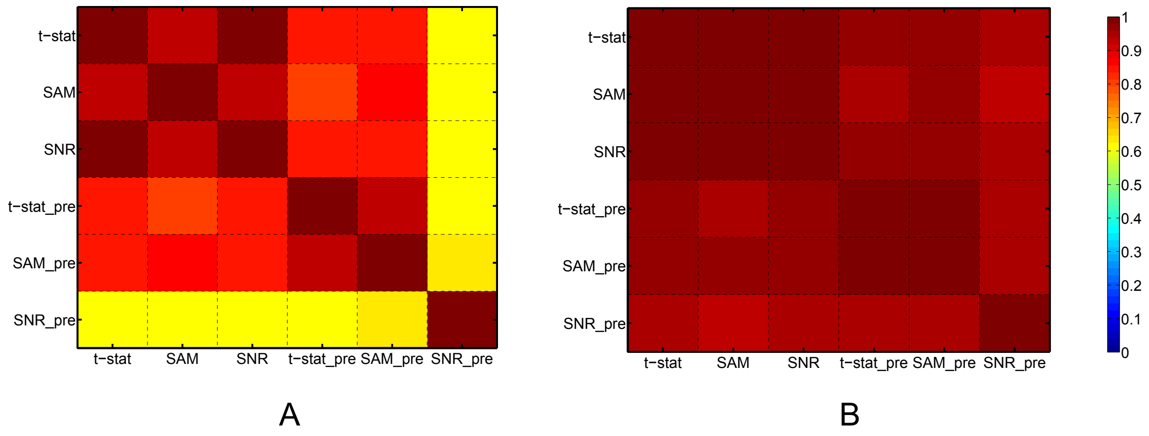

Figure S1: Correlation between different active score of genes. Subfigure A represents the results in the breast cancer metastasis dataset and subfigure B represents the results in the prostate cancer dataset.

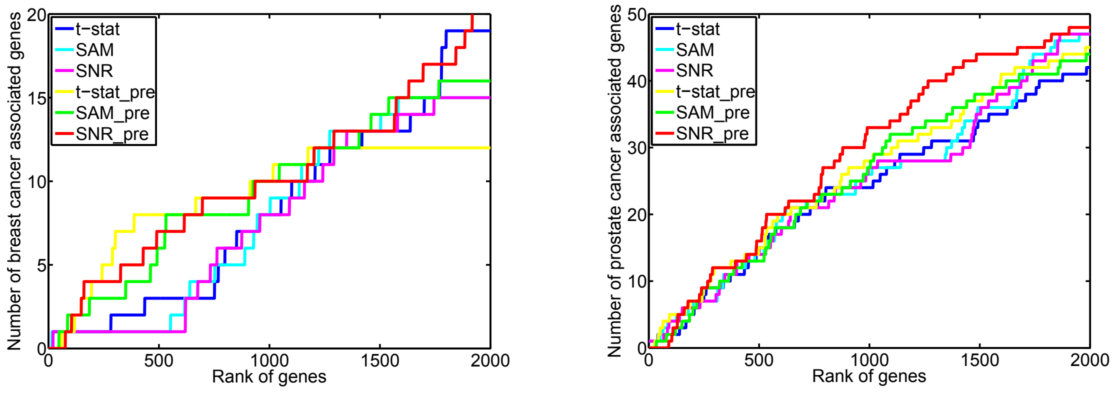

A B

Figure S2: The distribution of disease related gene in the ranking lists based on different active score of genes. Subfigure A represents the results in the breast cancer metastasis dataset and subfigure B represents the results in the prostate cancer dataset.

2 Sensitivity analysis

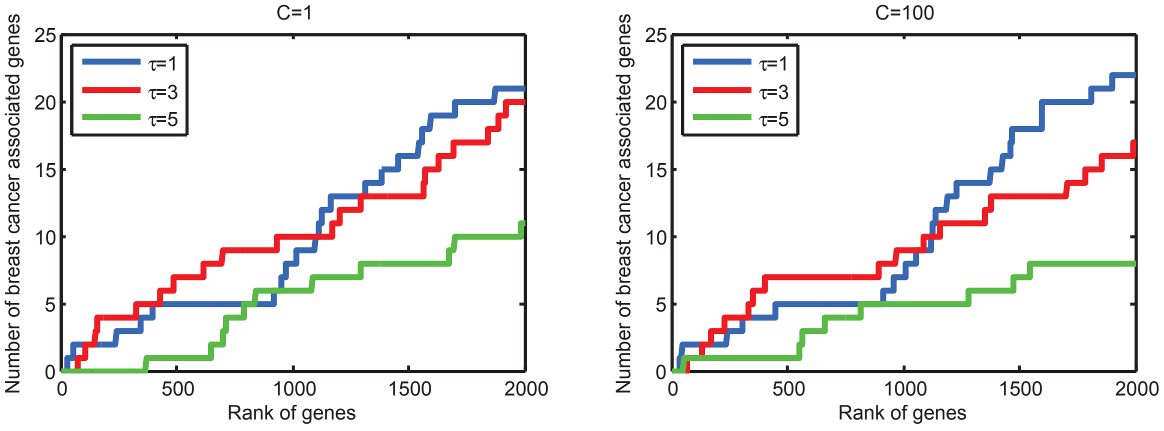

Figure S3: Parameter sensitivity analysis in breast cancer dataset. Using different combination of C and τ, the distribution of disease related gene in the ranking lists based on predicted active score of genes are compared.

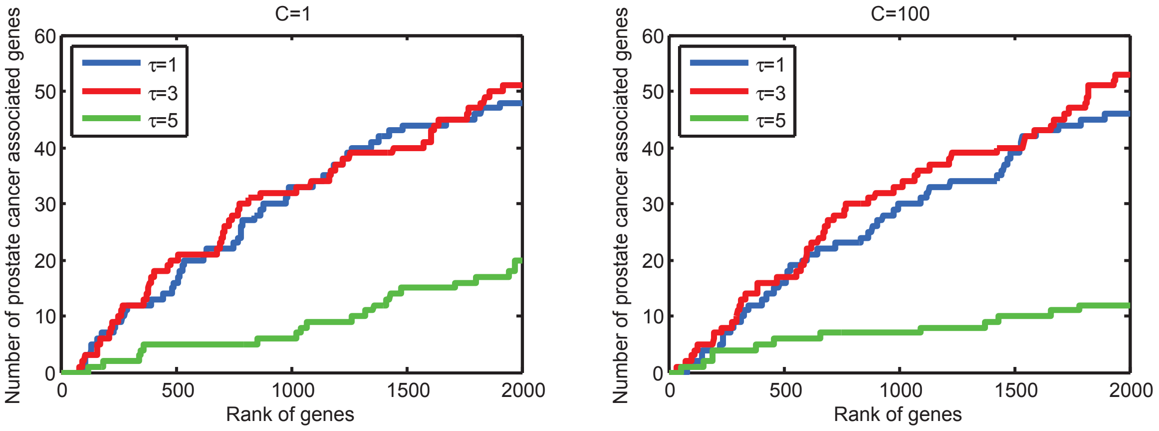

Figure S4: Parameter sensitivity analysis in prostate cancer dataset. Using different combination of C and τ, the distributions of disease related gene in the ranking lists based on predicted active score of genes are compared.

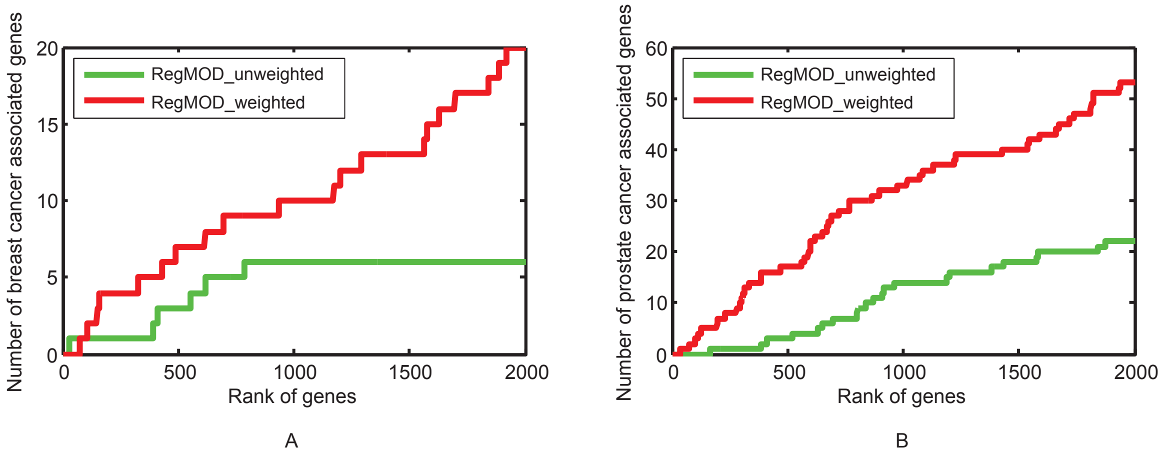

Figure S5: Comparison of RegMOD in terms of network with weighted and unweighted interaction. A is for the breast cancer dataset and B is for the prostate dataset. The parameters were all set that $C = 1$ and $\tau = 3$.

3 Threshold selection

The quality of the induced modules are evaluated by two quantitatively measures: cohesion s_m and average activity ac_m measurement. In the induced active modules including up-regulated and down-regulated genes, the cohesion which represents the within module similarity is calculated by mean value of similarity between each gene pair,

$$s_m = \frac{1}{|m|(|m|-1)} \sum_{i,j \in m, i \neq j} k_{ij},$$

where m represents a module and $|m|$ is the size of the module. The high cohesion indicates the genes within the module are more likely to function coordinately and to achieve one specific biological process. Thus, we should select the threshold to reveal the modules with high cohesion. Meanwhile, the active score of the modules is defined as the average underlying active score as follows,

$$ac_m = \frac{1}{|m|} \sum_{i \in m} f_i \quad .$$

These two measures guide us to select a proper θ to have consistent cohesion and high active score for further analysis. Specifically, we select $\theta = 6$ according to Figure S6.

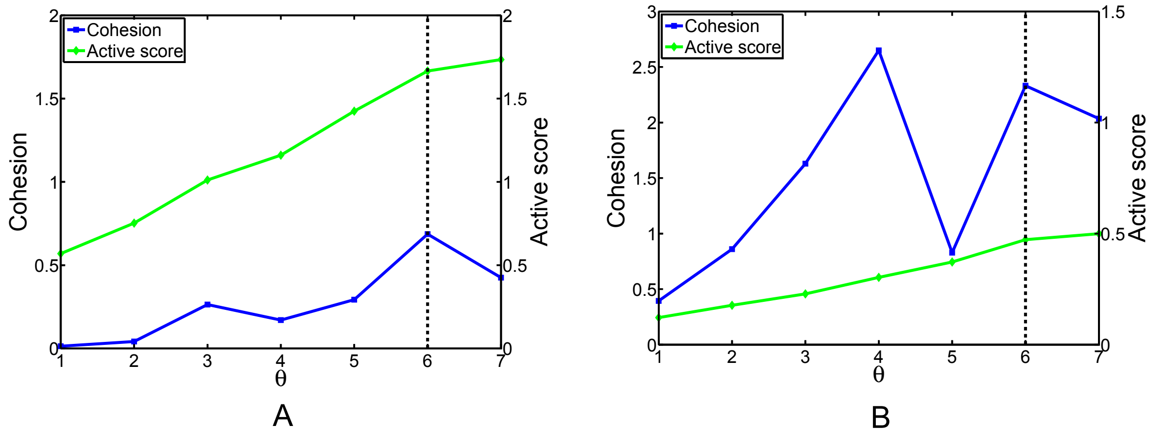

Figure S6: Quality of the active modules based on different threshold. Subfigure A represents the results in the breast cancer metastasis dataset and subfigure B represents the results in the prostate cancer dataset.

4 Note on the results of breast cancer dataset

The jActiveModules was used to find active subnetworks for comparison. Using the greedy search strategy of jActiveModules, we obtained 5 active subnetworks , and 3 of them contain more than 5 genes, denoted as BCjAM1, BCjAM2 and BCjAM3. But it outputted no active subnetworks when using the simulated annealing strategy. GiGA was also applied to these data by setting the parameter m to 52 and 33 for finding up- and down-regulated modules, and the result is compatible with the modules by our method. The identified 4 significant active subnetworks, i.e. 2 up- and 2 down-regulated modules which are denoted as BCGUM1, BCGUM2, BCGDM1 and BCGDM2 respectively.

Table S1: Comparison of modules' overlap of known pathways. The green cells represent pathways significantly overlapping with BCDM1, the red cells represent pathways significantly overlapping with BCDM2, and the blue cells represent pathways significantly overlapping with BCUM1.

RegMOD	*GiGA*	*jActiveModules*
BCDM1	BCGDM1	
IL6PATHWAY	TNFR2PATHWAY	
TPOPATHWAY	HSA04060 CYTOKINE CYTOKINE RECEPTOR INTERACTION	
APOPTOSIS KEGG	IL22BPPATHWAY	
EGFPATHWAY	IL7PATHWAY	
PDGFPATHWAY	RELAPATHWAY	
D4GDIPATHWAY	HSA04920 ADIPOCYTOKINE SIGNALING PATHWAY	
IL22BPPATHWAY	IL2RBPATHWAY	
HSA05223 NON SMALL CELL LUNG CANCER	HSA04630 JAK STAT SIGNALING PATHWAY	
IL2PATHWAY	HSA04640 HEMATOPOIETIC CELL LINEAGE	
CASPASEPATHWAY	BIOPEPTIDESPATHWAY	
PYK2PATHWAY	APOPTOSIS GENMAPP	
PKCPATHWAY	KERATINOCYTEPATHWAY	
HSA04920 ADIPOCYTOKINE SIGNALING PATHWAY	IL10PATHWAY	
STAT3PATHWAY	APOPTOSIS KEGG	
AT1RPATHWAY	THELPERPATHWAY	
IL2RBPATHWAY	HSA05221 ACUTE MYELOID LEUKEMIA	
BIOPEPTIDESPATHWAY	HIVNEFPATHWAY	
NUCLEAR RECEPTORS	41BBPATHWAY	
HSA04630 JAK STAT SIGNALING PATHWAY	CCR5PATHWAY	
	TIDPATHWAY	
BCDM2	BCGDM2	BCjAM2
HSA04612 ANTIGEN PROCESSING AND PRESENTATION	HSA04940 TYPE I DIABETES MELLITUS	HSA04612 ANTIGEN PROCESSING AND PRESENTATION
HSA04940 TYPE I DIABETES MELLITUS	HSA04612 ANTIGEN PROCESSING AND PRESENTATION	HSA04940 TYPE I DIABETES MELLITUS
HSA04514 CELL ADHESION MOLECULES	HSA04514 CELL ADHESION MOLECULES	HSA04514 CELL ADHESION MOLECULES
BBCELLPATHWAY	BBCELLPATHWAY	BBCELLPATHWAY
EOSINOPHILSPATHWAY	EOSINOPHILSPATHWAY	EOSINOPHILSPATHWAY
IL5PATHWAY	IL5PATHWAY	IL5PATHWAY
BLYMPHOCYTEPATHWAY	BLYMPHOCYTEPATHWAY	BLYMPHOCYTEPATHWAY
ASBCELLPATHWAY	ASBCELLPATHWAY	ASBCELLPATHWAY
TCRAPATHWAY	TCRAPATHWAY	TCRAPATHWAY
HSA04640 HEMATOPOIETIC CELL LINEAGE	TH1TH2PATHWAY	TH1TH2PATHWAY
TH1TH2PATHWAY	CTLA4PATHWAY	CTLA4PATHWAY
CTLA4PATHWAY	MTORPATHWAY	AMIPATHWAY
AMIPATHWAY	AMIPATHWAY	CSKPATHWAY
CSKPATHWAY	CSKPATHWAY	INFLAMPATHWAY
INFLAMPATHWAY	EIF4PATHWAY	HSA04640 HEMATOPOIETIC CELL LINEAGE
	INFLAMPATHWAY	
	HSA04640 HEMATOPOIETIC CELL LINEAGE	
BCUM1	BCGUM1	BCjAM1
HSA04110 CELL CYCLE	HSA04110 CELL CYCLE	CELL CYCLE KEGG
HSA05215 PROSTATE CANCER	CELL CYCLE KEGG	HSA04115 P53 SIGNALING PATHWAY
HSA04910 INSULIN SIGNALING PATHWAY	HSA04115 P53 SIGNALING PATHWAY	HSA04110 CELL CYCLE
HSA05212 PANCREATIC CANCER	DNA REPLICATION REACTOME	SA G2 AND M PHASES
MPRPATHWAY	CELLCYCLEPATHWAY	G2PATHWAY
HSA05220 CHRONIC MYELOID LEUKEMIA	G2PATHWAY	PTC1PATHWAY
HSA05214 GLIOMA	G1 TO S CELL CYCLE REACTOME	RBPATHWAY
HSA04720 LONG TERM POTENTIATION	PTC1PATHWAY	CELLCYCLEPATHWAY
HSA05211 RENAL CELL CARCINOMA	SRCRPTPPATHWAY	HSA05130 PATHOGENIC ESCHERICHIA COLI INFECTION EHEC
HSA05219 BLADDER CANCER	RBPATHWAY	HSA05131 PATHOGENIC ESCHERICHIA COLI INFECTION EPEC
HSA04930 TYPE II DIABETES MELLITUS	SA REG CASCADE OF CYCLIN EXPR	G1 TO S CELL CYCLE REACTOME
HSA04012 ERBB SIGNALING PATHWAY	P53PATHWAY	HIVNEFPATHWAY
RACCYCDPATHWAY	MPRPATHWAY	CDC25PATHWAY
HSA04350 TGF BETA SIGNALING PATHWAY	G1PATHWAY	DEATHPATHWAY
G2PATHWAY	HSA05215 PROSTATE CANCER	HSA05222 SMALL CELL LUNG CANCER
HSA05221 ACUTE MYELOID LEUKEMIA	PYRIMIDINE METABOLISM	MAPKPATHWAY
NFATPATHWAY	SIG PIP3 SIGNALING IN CARDIAC MYOCTES	APOPTOSIS
HSA05223 NON SMALL CELL LUNG CANCER	HSA04720 LONG TERM POTENTIATION	EPONFKBPATHWAY
	HSA05222 SMALL CELL LUNG CANCER	SRCRPTPPATHWAY
	HSA04350 TGF BETA SIGNALING PATHWAY	HSA05221 ACUTE MYELOID LEUKEMIA
BCUM2	BCGUM2	BCjAM3
ST FAS SIGNALING PATHWAY	PROTEASOME	HSA04620 TOLL LIKE RECEPTOR SIGNALING PATHWAY
	PROTEASOMEPATHWAY	TOLLPATHWAY
	HSA03050 PROTEASOME	GLEEVECPATHWAY
	GLUCONEOGENESIS	NGFPATHWAY
	GLYCOLYSIS	FCER1PATHWAY
	HSA00010 GLYCOLYSIS AND GLUCONEOGENESIS	IGF1PATHWAY
	HIFPATHWAY	INSULINPATHWAY
	GLYCOLYSIS AND GLUCONEOGENESIS	HSA05211 RENAL CELL CARCINOMA
		HSA04664 FC EPSILON RI SIGNALING PATHWAY
		EGFPATHWAY
		GSK3PATHWAY
		PDGFPATHWAY
		HSA04210 APOPTOSIS
		HSA05215 PROSTATE CANCER
		IL1RPATHWAY
		HSA04910 INSULIN SIGNALING PATHWAY
		AT1RPATHWAY
		LONGEVITYPATHWAY
		BCRPATHWAY
		HSA05214 GLIOMA

Table S2: Comparison of modules' enriched GO categories. The green cells represent GO categories enriched in BCDM1, the red cells represent GO categories enriched in BCDM2, and the blue cells represent GO categories enriched in BCUM1.

RegMOD	*GiGA*	*jActiveModules*
BCDM1	BCGDM1	
INTRACELLULAR SIGNALING CASCADE	JAK STAT CASCADE	
LIGAND DEPENDENT NUCLEAR RECEPTOR ACTIVITY	RECEPTOR ACTIVITY	
CYSTEINE TYPE ENDOPEPTIDASE ACTIVITY	PROTEIN KINASE CASCADE	
RESPONSE TO VIRUS	CYTOKINE BINDING	
STEROID HORMONE RECEPTOR ACTIVITY	PROTEIN TYROSINE KINASE ACTIVITY	
CYSTEINE TYPE PEPTIDASE ACTIVITY	TRANSMEMBRANE RECEPTOR ACTIVITY	
RECEPTOR ACTIVITY	MULTI ORGANISM PROCESS	
IMMUNE SYSTEM PROCESS	APOPTOSIS GO	
CELL SURFACE RECEPTOR LINKED SIGNAL TR TRANSDUCTION GO 0007166	PROGRAMMED CELL DEATH	
SECOND MESSENGER MEDIATED SIGNALING	SIGNAL TRANSDUCTION	
CELL CELL SIGNALING	IMMUNE SYSTEM PROCESS	
RESPONSE TO OTHER ORGANISM	CELL DEVELOPMENT	
EXOCYTOSIS	RESPONSE TO VIRUS	
PROTEIN KINASE CASCADE	PROTEIN KINASE ACTIVITY	
RESPONSE TO WOUNDING	TRANSCRIPTION FACTOR BINDING	
RESPONSE TO EXTERNAL STIMULUS	INTRACELLULAR SIGNALING CASCADE	
JAK STAT CASCADE	RESPONSE TO EXTERNAL STIMULUS	
G PROTEIN SIGNALING COUPLED TO CYCLIC NUCLEOTIDE SECOND MESSENGER	PROTEIN KINASE BINDING	
CYCLIC NUCLEOTIDE MEDIATED SIGNALING	ENZYME BINDING	
	PHOSPHOTRANSFERASE ACTIVITY ALCOHOL GROUP AS ACCEPTOR	
BCDM2	BCGDM2	BCjAM2
LYSOSOME	LYSOSOME	MEMBRANE
LYTIC VACUOLE	LYTIC VACUOLE	PLASMA MEMBRANE
VACUOLE	VACUOLE	
IMMUNE RESPONSE	INTRACELLULAR PROTEIN TRANSPORT	
IMMUNE SYSTEM PROCESS	PROTEIN TRANSPORT	
TRANSMEMBRANE RECEPTOR ACTIVITY	PROTEIN COMPLEX ASSEMBLY	
MEMBRANE		
RECEPTOR ACTIVITY		
PLASMA MEMBRANE		
INTEGRAL TO MEMBRANE		
INTRINSIC TO MEMBRANE		
CYTOPLASMIC PART		
MEMBRANE PART		
CYTOPLASM		
BCUM1	BCGUM1	BCjAM1
CELL CYCLE PROCESS	SPINDLE	CELL CYCLE GO 0007049
CELL CYCLE GO 0007049	CELL CYCLE PROCESS	CELL CYCLE PROCESS
MITOTIC CELL CYCLE	CELL CYCLE GO 0007049	MITOTIC CELL CYCLE
MITOTIC SPINDLE ORGANIZATION AND BIOGENESIS	MITOTIC CELL CYCLE	CELL CYCLE PHASE
SPINDLE ORGANIZATION AND BIOGENESIS	SPINDLE ORGANIZATION AND BIOGENESIS	NUCLEUS
CELL CYCLE PHASE	MICROTUBULE CYTOSKELETON	REGULATION OF CELL CYCLE
SPINDLE	SPINDLE MICROTUBULE	SPINDLE ORGANIZATION AND BIOGENESIS
MICROTUBULE CYTOSKELETON ORGANIZATION AND BIOGENESIS	CELL CYCLE PHASE	TRANSCRIPTION FACTOR BINDING
MICROTUBULE CYTOSKELETON	MITOTIC SPINDLE ORGANIZATION AND BIOGENESIS	SPINDLE
M PHASE	CYTOSKELETAL PART	TRANSCRIPTION COACTIVATOR ACTIVITY
MICROTUBULE BASED PROCESS	CYTOSKELETON	PHOSPHATASE REGULATOR ACTIVITY
M PHASE OF MITOTIC CELL CYCLE	MICROTUBULE	MITOSIS
INTRACELLULAR ORGANELLE PART	INTRACELLULAR NON MEMBRANE BOUND ORGANELLE	M PHASE OF MITOTIC CELL CYCLE
ORGANELLE PART	NON MEMBRANE BOUND ORGANELLE	SPINDLE MICROTUBULE
CYTOSKELETAL PART	MICROTUBULE CYTOSKELETON ORGANIZATION AND BIOGENESIS	M PHASE
SPINDLE MICROTUBULE	SPINDLE POLE	JAK STAT CASCADE
MITOSIS	M PHASE	PROTEIN COMPLEX
SPINDLE POLE	MICROTUBULE BASED PROCESS	TRANSCRIPTION ACTIVATOR ACTIVITY
CYTOSKELETON ORGANIZATION AND BIOGENESIS	REGULATION OF CELL CYCLE	MICROTUBULE CYTOSKELETON
INTRACELLULAR NON MEMBRANE BOUND ORGANELLE	M PHASE OF MITOTIC CELL CYCLE	REGULATION OF PROTEIN KINASE ACTIVITY
BCUM2		BCjAM3
INDUCTION OF APOPTOSIS BY EXTRACELLULAR SIGNALS		POSITIVE REGULATION OF CYTOKINE BIOSYN SYNTHETIC PROCESS
REGULATION OF APOPTOSIS		POSITIVE REGULATION OF TRANSLATION
REGULATION OF PROGRAMMED CELL DEATH		REGULATION OF CYTOKINE BIOSYNTHETIC PROCESS
APOPTOSIS GO		CYTOKINE BIOSYNTHETIC PROCESS
PROGRAMMED CELL DEATH		POSITIVE REGULATION OF CELLULAR PROTEIN METABOLIC PROCESS
REGULATION OF DEVELOPMENTAL PROCESS		CYTOKINE METABOLIC PROCESS
CELL DEVELOPMENT		POSITIVE REGULATION OF PROTEIN METABOLIC PROCESS
POSITIVE REGULATION OF DEVELOPMENTAL PROCESS		REGULATION OF CELLULAR PROTEIN METABOLIC PROCESS
IDENTICAL PROTEIN BINDING		I KAPPAB KINASE NF KAPPAB CASCADE
RECEPTOR BINDING		CYTOKINE PRODUCTION
INTRACELLULAR SIGNALING CASCADE		REGULATION OF PROTEIN METABOLIC PROCESS
POSITIVE REGULATION OF CELLULAR PROCESS		POSITIVE REGULATION OF CELLULAR PROCESS
POSITIVE REGULATION OF BIOLOGICAL PROCESS		REGULATION OF I KAPPAB KINASE NF KAPPAB CASCADE
NUCLEUS		REGULATION OF TRANSLATION
SIGNAL TRANSDUCTION		POSITIVE REGULATION OF BIOLOGICAL PROCESS
CYTOPLASM		PROTEIN KINASE CASCADE
		POSITIVE REGULATION OF CELLULAR METABOLIC PROCESS
		POSITIVE REGULATION OF METABOLIC PROCESS
		MACROMOLECULE BIOSYNTHETIC PROCESS
		LIPID RAFT

5 Note on the results of prostate cancer dataset

The GiGA found 373 genes involving 2 up- and 2 down-regulated modules, denoted as PCGUM1 PCGUM2, PCGDM1 and PCGDM2. The parameter m used in GiGA was set to 69 which is the size of PCDM1. The jActiveModules found an active module of 441 genes, denoted as PCjAM1.

Table S3: Comparison of modules' overlap of known pathways. The green cells represent pathways significantly overlapping with PCDM1, the red cells represent pathways significantly overlapping with PCDM2, and the blue cells represent pathways significantly overlapping with PCDM3.

RegMOD	*GiGA*	*jActivModules*
PCDM1	PCGDM1	PCjAM1
HSA04520_ADHERENS_JUNCTION	HSA05220_CHRONIC_MYELOID_LEUKEMIA	HSA04510_FOCAL_ADHESION
ERYTHPATHWAY	HSA05212_PANCREATIC_CANCER	HSA04520_ADHERENS_JUNCTION
TOB1PATHWAY	HSA04520_ADHERENS_JUNCTION	HSA05212_PANCREATIC_CANCER
HSA04510_FOCAL_ADHESION	HSA05210_COLORECTAL_CANCER	PYK2PATHWAY
HSA04060_CYTOKINE_CYTOKINE_RECEPTOR_INTERACTION	TOB1PATHWAY	ST_INTEGRIN_SIGNALING_PATHWAY
HSA05220_CHRONIC_MYELOID_LEUKEMIA	HSA04510_FOCAL_ADHESION	HSA05220_CHRONIC_MYELOID_LEUKEMIA
METPATHWAY	BADPATHWAY	METPATHWAY
ALKPATHWAY	RAC1PATHWAY	CXCR4PATHWAY
INTEGRIN_MEDIATED_CELL_ADHESION_KEGG	TPOPATHWAY	HSA04810_REGULATION_OF_ACTIN_CYTOSKELETON
BIOPEPTIDESPATHWAY	HSA04060_CYTOKINE_CYTOKINE_RECEPTOR_INTERACTION	HSA04012_ERBB_SIGNALING_PATHWAY
CARDIACEGFPATHWAY	EGFPATHWAY	MAPKPATHWAY
HSA04916_MELANOGENESIS	HSA04630_JAK_STAT_SIGNALING_PATHWAY	EGFPATHWAY
HSA05211_RENAL_CELL_CARCINOMA	IL10PATHWAY	INTEGRINPATHWAY
TGFBPATHWAY	IL22BPPATHWAY	RACCYCDPATHWAY
HSA05212_PANCREATIC_CANCER	HSA04012_ERBB_SIGNALING_PATHWAY	TPOPATHWAY
IL6PATHWAY	ERYTHPATHWAY	BIOPEPTIDESPATHWAY
HSA04630_JAK_STAT_SIGNALING_PATHWAY	HSA04010_MAPK_SIGNALING_PATHWAY	AT1RPATHWAY
TPOPATHWAY	HSA04810_REGULATION_OF_ACTIN_CYTOSKELETON	HSA05215_PROSTATE_CANCER
ST_INTEGRIN_SIGNALING_PATHWAY	METPATHWAY	INTEGRIN_MEDIATED_CELL_ADHESION_KEGG
	IL7PATHWAY	FMLPPATHWAY
PCDM2	PCGDM2	
HSA04810_REGULATION_OF_ACTIN_CYTOSKELETON	HSA04520_ADHERENS_JUNCTION	
HSA04010_MAPK_SIGNALING_PATHWAY	CELL2CELLPATHWAY	
HSA04512_ECM_RECEPTOR_INTERACTION	INTEGRINPATHWAY	
HSA04510_FOCAL_ADHESION	INTEGRIN_MEDIATED_CELL_ADHESION_KEGG	
	HSA04670_LEUKOCYTE_TRANSENDOTHELIAL_MIGRATION	
	HSA01430_CELL_COMMUNICATION	

Table S4: Comparison of modules' enriched GO categories. The green cells represent GO categories enriched in PCDM1, the red cells represent GO categories enriched in PCDM2, and the blue cells represent GO categories enriched in PCDM3.

RegMOD	*GiGA*	*jActivModules*
PCDM1	PCGDM1	PCjAM1
CELL_MATRIX_JUNCTION	SIGNAL_TRANSDUCTION	CYTOSKELETON
BASOLATERAL_PLASMA_MEMBRANE	TRANSMEMBRANE_RECEPTOR_PROTEIN_KINASE_ACTIVITY	ACTIN_FILAMENT_BASED_PROCESS
CELL_JUNCTION	POSITIVE_REGULATION_OF_CELLULAR_PROCESS	POSITIVE_REGULATION_OF_CELLULAR_PROCESS
CELL_SUBSTRATE_ADHERENS_JUNCTION	POSITIVE_REGULATION_OF_BIOLOGICAL_PROCESS	BLOOD_COAGULATION
ADHERENS_JUNCTION	RECEPTOR_COMPLEX	CYTOSKELETAL_PROTEIN_BINDING
FOCAL_ADHESION_FORMATION	POSITIVE_REGULATION_OF_CELL_PROLIFERATION	INTRINSIC_TO_MEMBRANE
SIGNAL_TRANSDUCTION	INTRACELLULAR_SIGNALING_CASCADE	CYTOSKELETON_ORGANIZATION_AND_BIOGENESIS
FOCAL_ADHESION	PROTEIN_KINASE_ACTIVITY	COAGULATION
POSITIVE_REGULATION_OF_CELLULAR_PROCESS	PROTEIN_TYROSINE_KINASE_ACTIVITY	REGULATION_OF_CELL_MIGRATION
CELL_MATRIX_ADHESION	JAK_STAT_CASCADE	WOUND_HEALING
CELL_SUBSTRATE_ADHESION	TRANSFORMING_GROWTH_FACTOR_BETA_RECEPTOR_SIGNALING_PATHWAY	MEMBRANE_PART
REGULATION_OF_PROTEIN_IMPORT_INTO_NUCLEUS	PHOSPHOTRANSFERASE_ACTIVITY__ALCOHOL_GROUP_AS_ACCEPTOR	POSITIVE_REGULATION_OF_BIOLOGICAL_PROCESS
POSITIVE_REGULATION_OF_BIOLOGICAL_PROCESS	RECEPTOR_SIGNALING_PROTEIN_ACTIVITY	INTEGRAL_TO_MEMBRANE
PROTEIN_IMPORT_INTO_NUCLEUS	SMAD_BINDING	ACTIN_CYTOSKELETON
NUCLEAR_IMPORT	ENZYME_LINKED_RECEPTOR_PROTEIN_SIGNALING_PATHWAY	CELL_SUBSTRATE_ADHERENS_JUNCTION
REGULATION_OF_NUCLEOCYTOPLASMIC_TRANSPORT	TRANSMEMBRANE_RECEPTOR_PROTEIN_TYROSINE_KINASE_ACTIVITY	SUBSTRATE_SPECIFIC_TRANSPORTER_ACTIVITY
RECEPTOR_COMPLEX	G_PROTEIN_SIGNALING__COUPLED_TO_IP3_SECOND_MESSENGER__PHOSPHOLIPASE_C_ACTIVATING	HEMOSTASIS
REGULATION_OF_INTRACELLULAR_TRANSPORT	PHOSPHOLIPASE_C_ACTIVATION	CYTOSKELETAL_PART
REGULATION_OF_CELL_MIGRATION	TRANSMEMBRANE_RECEPTOR_PROTEIN_SERINE_THREONINE_KINASE_SIGNALING_PATHWAY	NEGATIVE_REGULATION_OF_METABOLIC_PROCESS
RESPONSE_TO_HYPOXIA	KINASE_ACTIVITY	INTRACELLULAR_NON_MEMBRANE_BOUND_ORGANELLE
PCDM2	PCGDM2	
RECEPTOR_BINDING	CELL_MATRIX_JUNCTION	
	CELL_JUNCTION	
	BASOLATERAL_PLASMA_MEMBRANE	
PCDM3	CELL_SUBSTRATE_ADHERENS_JUNCTION	
REGULATION_OF_APOPTOSIS	ADHERENS_JUNCTION	
REGULATION_OF_PROGRAMMED_CELL_DEATH	FOCAL_ADHESION	
NERVOUS_SYSTEM_DEVELOPMENT	CYTOSKELETAL_PROTEIN_BINDING	
APOPTOSIS_GO	CELL_MATRIX_ADHESION	
PROGRAMMED_CELL_DEATH	CELL_SUBSTRATE_ADHESION	
REGULATION_OF_DEVELOPMENTAL_PROCESS	PROTEIN_COMPLEX_BINDING	
EXTRACELLULAR_REGION	FOCAL_ADHESION_FORMATION	
CELL_DEVELOPMENT	INTERCELLULAR_JUNCTION	
STRUCTURAL_MOLECULE_ACTIVITY	ACTIN_BINDING	
SYSTEM_DEVELOPMENT	CYTOSKELETAL_PART	
EXTRACELLULAR_REGION_PART	MYOFIBRIL	
ANATOMICAL_STRUCTURE_DEVELOPMENT	CONTRACTILE_FIBER_PART	
MULTICELLULAR_ORGANISMAL_DEVELOPMENT	CONTRACTILE_FIBER	
PROTEIN_METABOLIC_PROCESS	REGULATION_OF_CELL_MIGRATION	
	ACTIN_CYTOSKELETON	
	INTEGRIN_BINDING	
